# Implementing a complex innovation into workplace operations: The case of work ability support for indoor environment-associated health complaints

**DOI:** 10.1177/10519815251414377

**Published:** 2026-02-02

**Authors:** Hanna Keränen, Aki Vuokko, Pirjo Korenius, Sanna Selinheimo, Pirjo Juvonen-Posti

**Affiliations:** 1 Work Ability and Working Careers, Finnish Institute of Occupational Health, Oulu, Finland; 2 Occupational Health, Finnish Institute of Occupational Health, Helsinki, Finland; 3 Occupational Health, Finnish Institute of Occupational Health, Kuopio, Finland; 4 Work Ability and Working Careers, Finnish Institute of Occupational Health, Helsinki, Finland

**Keywords:** implementation science, indoor air pollution, occupational health, workplace, preventive health services, working conditions

## Abstract

**Background:**

Multifactorial health complaints associated with indoor work environments can impact on employees’ work ability and arouse controversies in the workplace. Traditionally, solutions have centered on building-related factors, however supporting work ability requires a multi-perspective approach.

**Objective:**

This study explored relevant perspectives for implementing a multi-perspective work ability support model. This model represents a complex intervention within the intricate context of indoor environment-related health complaints in the workplace.

**Methods:**

We used recordings and notes from the workshops of a project aiming to implement the work ability support model in three organizations and used reflexive thematic analysis. We developed overarching themes from the data, using the Consolidated Framework for Implementation Research (CFIR) as an interpretative lens to structure the findings into implementation perspectives.

**Results:**

We developed four overarching themes encompassing all CFIR domains and most constructs: (1) the acknowledgment and validation of employees’ emotions and experiences as a solution, (2) reliable information and detailed facts as the key to managing work ability support, (3) existing tools as a means to normalization and collaboration, and (4) supervisors as central actors in a challenging task.

**Conclusions:**

Implementing a work ability support model regarding complex phenomena affecting work ability, such as indoor environment-related health complaints, requires identifying and addressing the potential tensions and controversies underlying the model and requires consideration of the context. Viewing communication as a process of developing shared understanding and co-constructing knowledge throughout the implementation process and all its dimensions can be beneficial for the implementation.

## Introduction

Managing various illnesses and related disabilities that impair work performance has become increasingly important in both workplace and healthcare settings, driven by the pressure to extend working careers. Health complaints emerging from multifactorial origin, which cannot be explained by traditional diseases or environmental factors, are prevalent, vary across countries, and pose significant challenges to work ability support measures, particularly when prolonged.^1,2^ These complaints are associated with decreased labor market participation, including long-term sickness absences and unemployment, and may increase the risk of disability pension.^3,4^ When integrating work modifications with individual care, the work-oriented approach was more effective than focusing solely on individual treatment and may reduce sickness absences among individuals with prolonged health issues.^5^ This study focuses on work ability support in the case of multifactorial health complaints that are particularly associated with indoor environments.

Workplace programs targeting disability management and reducing sickness absences are considered efficient and effective,^6^ with many organizations integrating these programs into their management processes. Supporting work ability involves complex interventions that feature multiple interacting components, target diverse groups and behaviors, produce various outcomes, or require tailoring to specific contexts.^7,8^ These processes engage various actors within the workplace and in other settings, such as occupational healthcare and rehabilitation settings. The diverse interests of stakeholders can create friction, necessitating strategies that reflect all stakeholders’ interests and determine the optimal level and timing of their involvement.^9^ The effects of work ability support interventions arise from complex causal chains with intertwined promoting and preventing factors.^10–12^ These support processes are seen as social interventions within complex systems, producing effects through intricate networks.^11,13^ This underscores the importance of considering the context.^7,14^

Our study examines the implementation of the recently developed work ability support model for managing health complaints associated with indoor work environments.^15^ This model features many aspects of a complex intervention, approaching indoor environment-related health complaints from multiple perspectives and engaging various stakeholders in work ability support.^15^ In Finland, where our study is based, employers are legally obligated to support employees’ work ability.^16^ This obligation has led to widespread adaptation of supportive measures within organizations. However, these measures often occur too late in cases of health complaints associated with indoor environments, as attempts to resolve these issues have traditionally focused on the building itself.^15^ Furthermore, the implementation of this complex intervention is further challenged by both the intricate nature of the targeted health phenomenon and its controversial context, which will be elaborated in the following section.

### The complex and controversial case of work ability support for health complaints associated with indoor environments

Health complaints attributed to indoor environments are an inherently intricate phenomenon, as they are subjective and multifactorial, usually linked to non-industrial settings with pollutant levels far below toxic thresholds, and emerging as a public health concern.^17–20^ These complaints typically manifest as non-specific symptoms within certain buildings and range from mild discomfort to disabling conditions in some individuals.^18,19^ Their multifactorial nature involves the interaction and coexistence building-related factors, individual characteristics, and psychosocial elements.^19^ Thus, they have a biopsychosocial nature rather than being purely organically based and explained by toxicological responses, and they also may result in the avoidance of specific situations that are perceived to trigger symptoms.^2,21–23^ These health complaints impact on daily life, the quality of life, work attendance, and productivity of persons suffering from them.^23–25^ The interplay between symptom awareness and risk perceptions related to indoor environments can create a vicious circle, negatively affecting personal, work, and social life.^25,26^

Previous research shows that interventions for multifactorial and prolonged health complaints typically require a multi-perspective approach.^27,28^ Additionally, a biopsychosocial approach is recommended when preventing indoor environment-related disabilities,^24,29^ as it considers the holistic impact of work on physical, psychological, and social well-being.^5^ The model implemented in this study employs a multi-perspective approach, involving multiple stakeholders and multiple components, meaning it is a complex intervention.^8,10^

In addition to the model being a complex intervention, the implementation context is also complex. In Finland, health complaints associated with indoor environments have led to public controversy, focusing on whether these issues stem from building-related or psychosocial factors.^30–32^ Despite both these perspectives being overly reductive, given that scientific research has demonstrated the multifactorial nature of these complaints, building-focused views persist among the public and some researchers.^30–32^ Concurrently, in medical research, these multifactorial health complaints have previously been labeled as “medically unexplained” due to a lack of exposure-related or disease-related explanations and objectively verifiable signs.^23,27^ Thus, these complaints have challenged the epistemology of biomedicine and created epistemic tensions and controversy.^33^

Reflecting this controversy, the medical evaluation process in Finland further exemplifies the complexity of addressing health complaints linked to indoor environments. Asthma induced by indoor air molds was classified as an occupational disease in the 1990s under the biomedical framework, yet research has not established a causal link between mold exposure and asthma.^34^ To investigate the possibility of an occupational asthma induced by indoor mold exposure, patients are referred to specialized clinics in Finland for medical examinations according to nationally agreed criteria.^34,35^ These examinations aim to systematically assess potential connections between symptoms and environmental factors.^34,35^ However, patients often present with non-specific symptoms that extend beyond typical asthma features, paralleling the multifactorial and subjective nature highlighted in the public debate. These symptoms can contribute to disabilities and necessitate more intensive interventions.^23,34,35^

In summary, the implementation of a model designed to support the work ability of employees who experience health complaints associated with indoor work environments represents a complex intervention that has the potential to spark controversy. The complexity arises from the need to address the subjective and multifactorial nature of these health complaints, which may involve various factors such as environmental, psychosocial, and individual health aspects. To our knowledge, there is no previous research that has explored the implementation of such a comprehensive work ability support intervention for subjective health complaints in workplaces, especially within a context that is already marked by public debate and differing perceptions.

The findings of the study may contribute to the implementation research of complex interventions by examining the implementation of an intricate health phenomenon in a controversial context. As our overarching themes will show, tensions arising from the phenomenon's complexity and controversiality may affect various implementation aspects widely. As we will show, implementing a complex intervention addressing an intricate health phenomenon may require viewing communication as a process of developing shared understanding and co-constructing knowledge throughout the implementation process and all its dimensions, instead of a merely transactional process.

This study aims to answer the following research questions: (i) What perspectives do participants consider relevant for implementing this work ability support model in a potentially controversial context? (ii) What consequences do these have for the implementation? By addressing these questions, we aim to enhance understanding of implementing complex interventions in controversial contexts, as both complexity and controversy may impact various aspects of implementation.

## Data and methods

### Study design

This study was part of a research and development project by Finnish Institute of Occupational Health, focused on implementing a work ability support model for addressing health complaints associated with indoor environments.^15^ The ethics approval was obtained from the ethics committee of the Finnish Institute of Occupational Health (ETR 01/2022 on 16 March 2022), and participants provided their written informed consent. Data collection began only after the ethics approval was obtained. The project timeline and the data collection process for this study are illustrated in Table 1, which also presents the themes and discussion prompts of each workshop.

**Table 1.** The project timeline and the data collection process from the baseline to seven months.

Baseline	1.5 months	5.0 months	6.5 months	7.0 months
**Workshop 1** (3.0 h)	**Workshop 2** (2.0 h)	**Workshop 3** (2.5 h)	**Workshop 4** (1.0 h)	**Workshop 5** (2.0 h)
Theme and form				
Opening, inter-organizational		Communication, inter-organizational		Closing, inter-organizational
	Planning, organization 1		Implementation support, organization 1	
	Planning, organization 2		Implementation support, organization 2	
	Planning, organization 3		Implementation support, organization 3	
Contents and data				
- Participant introductions - Presentation: Vision for the work ability support model - Group discussion: What aspects of the model would I like to implement at my workplace immediately? (*Recordings, transcripts, notes*) - Presentation: Methods used in the project, introduction of the Planning phase checklist - Small group work and collective discussion: Defining the organization's change goal (*Recordings, transcripts, notes*) - Assignment: Continue clarifying the change goal and introduction to the next workshop	- Group discussion: Structuring the organization's change goal. What concrete operational change should take place in supporting work ability in your organization in indoor air situations? (*Notes*) - Collaborative work: Using the Planning phase checklist to identify actions required for the change (*Notes*) - Presentation: Introduction to the next workshop and the Communication tool	- Brief presentations: Progress and the phase of change goal for each organization (*Notes*) - Presentation: Introduction of the Communication tool - Small group work and collective discussion: Reflection on individuals and groups affected by the change goal (*Notes*) - Collaborative work: An in-depth review of a selected personnel group as an example (*Notes*) - Presentation: Introduction of the Implementation support tool	- Collaborative work: Completing the Implementation support tool (*Notes*) - Assignment: Preparation for the closing workshop	- Presentations by organizations: The change goal and its refinement during the project. Experiences from the project and the tools used. The benefits and challenges of the development work (*Recordings, transcripts, notes*) - Presentation: A summary of project progress and the tools used. Sources of further information. - Group discussion: A concrete timeline for progress. What will you do in the next month and six months? (*Recordings, transcripts, notes*)

**Figure 1.**
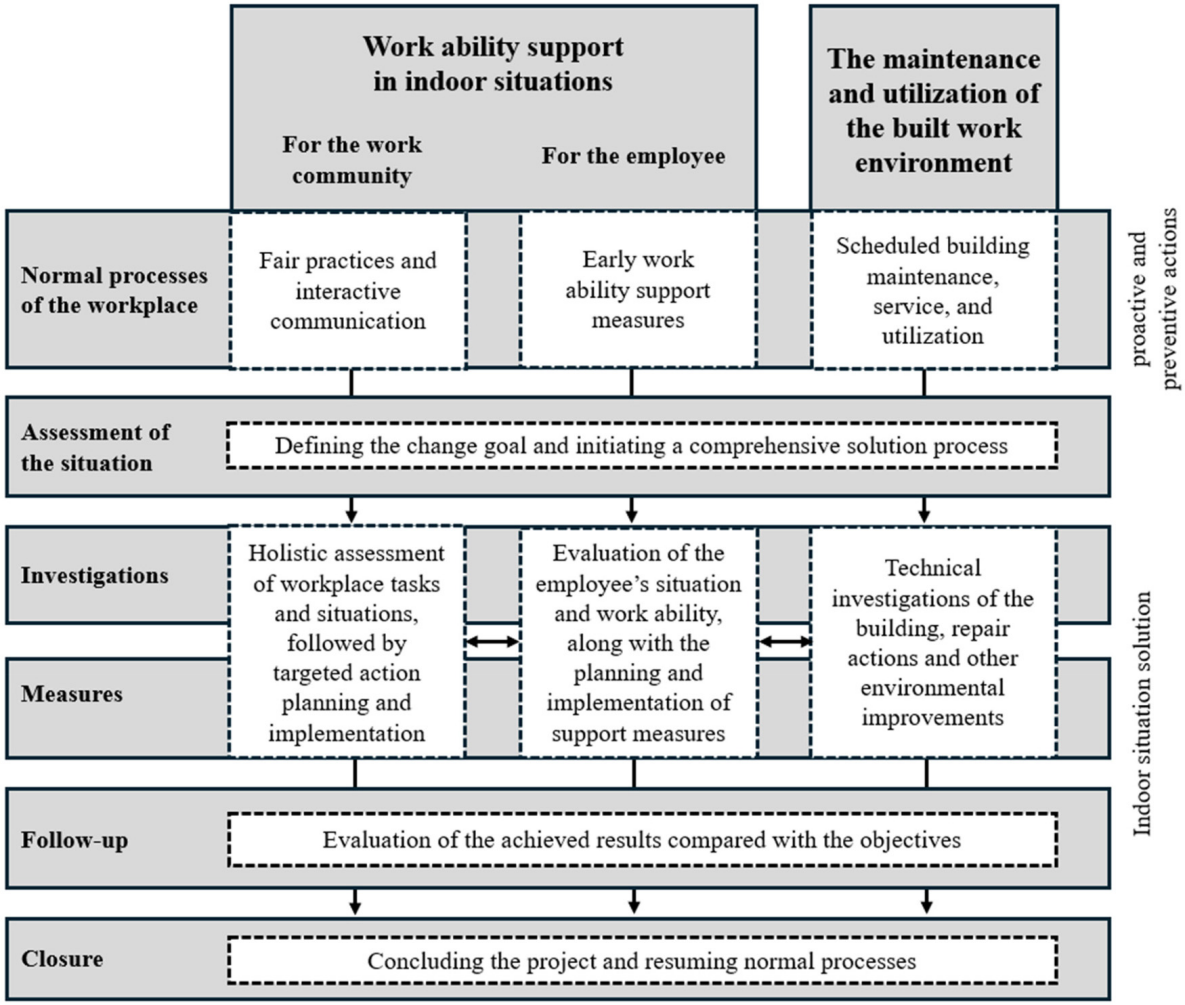
The work ability support model for managing indoor environment-related health complaints in the workplace.^15^.

The model for work ability support for indoor environment-related health complaints that was implemented in this study integrates work ability support processes and practices with the maintenance and improvement of building conditions.^15^ It emphasizes preventive measures and a process-oriented approach, and the main idea of the model is to detect employees’ work ability support needs in an early stage, when indoor environment-related health complaints are reported (Figure 1).

Figure 1 illustrates two intertwined processes: a building-related process and a work ability support process, which is further divided into support for the individual worker and for the work community. The key aspect of the model is the simultaneous advancement of these processes, addressing both building issues and work ability issues concurrently. The first level, the normal processes of the workplace, emphasizes the importance of having day-to-day workplace processes well-established, so that if an indoor environment issue arises, the response can be more efficient and effective. The second level outlines the joint assessment and goal-setting phase among workplace actors when an indoor environment issue is suspected. Following this, the agreed-upon investigations, measures, and subsequent follow-up actions take place. The model also emphasizes the importance of a concluding phase, as indoor environment processes often become unnecessarily prolonged and lack a proper resolution.

We employed the Consolidated Framework for Implementation Research (CFIR) in planning the overall research and development process, structuring the contents of the workshops, and analyzing our research data. The CFIR integrates multiple implementation theories into a comprehensive typology, encompassing five key domains: Innovation (i.e., intervention), Outer Setting, Inner Setting, Individuals, and Implementation Process, each with several constructs that describe factors influencing implementation.^36,37^ Although the development work by participating organizations followed a timeline presented in Table 1, our study did not aim to examine follow-up changes.

### Study participants

Organizations that had participated in previous development projects related to indoor environment themes or had developed work ability support practices at their workplaces, were contacted for recruitment into the study using purposive sampling. Several organizations expressed interest in participating, and the first three were selected, representing the sectors of childhood and education, security, and social care and healthcare. The project's emphasis on in-depth facilitation and support required limiting the number of participating organizations, which also enabled more meaningful discussions during the workshops.

The organizations were invited to assemble a team to participate in the project. Each organization's team served as its own implementation team, actively participating in workshops and collaborating on the planning and execution of the implementation. Team compositions varied based on organizational needs and goals but included members representing both indoor environment processes and work ability support processes. The number of team members ranged from 4 to 7, totaling 16 participants in this study. Participants’ occupational roles included management (e.g., occupational safety managers, work environment managers and upper-level managers), HR representatives (e.g., work ability support specialists and occupational safety specialists), occupational healthcare representatives, and health and safety representatives as representing employees.

Further demographic information was not collected for the following reasons. As we will elaborate in subsection 2.4, which discusses the analytical approach and process, our approach in this study was constructionist. Constructionist approach emphasizes how meanings are constructed and interpreted within the data, rather than explaining patterns through background variables. Although demographic information can provide context, the themes are developed across the entire dataset, rather than being determined by participant characteristics. Therefore, demographic details are not essential for ensuring analytic quality or shaping themes.^38^

### Data

As Table 1 illustrates, our data comprised of workshop recordings, transcriptions and notes. The data were collected from March to November in 2022. The primary data source was recordings from two workshops, totaling 191 min, which were transcribed verbatim. We utilized both recordings and transcriptions in analyzing the data to ensure accuracy. With a constructionist approach, we focused on capturing implementation team members’ perceptions, making recorded workshop conversations ideal for representing participants’ voices.^38^ Three other workshops (see Table 1) were observed by two researchers who took notes. These notes were not directly analyzed but were used to enhance understanding of the implementation process and support interpretations from the recorded data. Data saturation was not pursued in the study, because it is not relevant or theoretically coherent considering the context-dependent and interpretative nature of our analysis method, reflexive thematic analysis (RTA).^38^

### The analytical approach and process

We utilized both deductive coding using an existing theoretical framework (CFIR) to contextualize the data excerpts, and a more purely data-driven, inductive approach to explore aspects of the implementation and its context regarding the work ability support model. This involved both deductive and inductive phases in the analysis. We chose reflexive thematic analysis (RTA) as our method which, while primarily inductive, permits the use of theoretical frameworks as interpretative lenses.^39^ RTA is also inherently flexible, guiding researchers to articulate their theoretical assumptions and choices in the research process.^40,41^ Given our interest in exploring the implementation team members’ perceptions, RTA with a constructionist approach allowed us to delve into participants’ own meanings and what they considered meaningful.^42^ Essentially, we focused on the team members’ subjective experiences and meanings and their implications for the implementation process, which can be seen as arising from social interactions and (organizational) culture.^43^

Our analytical process began with a thorough review of the recordings, transcriptions, and observation notes to familiarize ourselves with the data. The first author then conducted a deductive coding phase, serving as a preliminary mapping for results obtained in the subsequent inductive phase. Using the CFIR as guidance, the first author identified meaning units relevant to the implementation, with units often fitting multiple categories. Although CFIR provides detailed definitions for each construct,^36^ it was used as a guide to structure meaning units into various implementation perspectives rather than strictly forcing data to domains and constructs. We defined meaning units as segments of speech—either a turn of speech or part of a turn—since our primary data consisted of recordings and transcripts of workshop conversations.

After coding all meaning units according to CFIR domains and constructs, we initiated the inductive phase of analysis. This data-driven phase partially overlapped with the deductive phase and focused on deepening the analysis of meaning units through latent coding, or interpretative coding, to uncover underlying meanings, such as assumptions, ideas, and concepts, rather than merely examining the directly expressed semantic contents in the data. The objective of this phase was to identify overarching themes meaningful for the participants within the implementation process and its context. Four authors (HK, AV, PK, PJ-P) collaborated in this phase, conducting data analysis sessions to code, develop and iteratively refine initial themes.^41^ During the writing process, theme names were adjusted to accurately reflect their dimensions. The research team engaged in reflexive discussions to interpret findings throughout each analysis phase.

Finally, we aligned each overarching theme with the CFIR domains and constructs using our preliminary deductive coding results. The goal of the deductive phase was to help understand and contextualize participants’ perspectives in relation to CFIR, without aiming to assess the validity of their views. In this way, the deductive phase established a broader context to which subsequent inductive analysis deepened. The goal was to demonstrate the extent to which each inductively developed, overarching theme encompasses the domains and constructs within the CFIR framework. An overall chart of the findings is presented in the Results section.

The research team worked reflexively, discussing our findings and interpretations in each phase of the analytical process. All the authors hold clinical degrees: HK, AV, PK, and PJ-P are medical doctors, while SS is a clinical psychologist. We have all previously contributed to research on health complaints associated with indoor environments. PJ-P has experience of implementation research, work ability support at the workplace, and work-related rehabilitation. HK, AV, and PJ-P have previous experience of qualitative research.

## Results

In this section, we present the results of our analysis. Through deductive coding, we identified implementation aspects viewed as meaningful by the participants, examining these meanings in relation to the CFIR framework. Our data encompassed all CFIR domains, but not all the domains’ constructs in the updated CFIR.^37^ In the subsequent inductive phase, we developed four themes reflecting the contextual aspects of implementing a work ability support model for complex health issues. The themes, along with related CFIR domains and constructs, are detailed in Table 2 and elaborated on using representative excerpts in the following subsections. Examples of quotations for each theme are presented under corresponding subsections.

**Table 2.** The CFIR domains and constructs associated with qualitative themes.

Theme	CFIR domain	CFIR constructs
1: The acknowledgment and validation of employees’ emotions and experiences as a solution	Innovation	Relative Advantage, Adaptability, Trialability, Complexity*
	Outer Setting	Partnerships & Connections*, Policies & Laws
	Inner Setting	Structural Characteristics*, Relational Connections, Communications*, Culture*, Compatibility
2: Reliable information and detailed facts as the key to managing work ability support	Innovation	Source
	Outer Setting	Local Attitudes
	Inner Setting	Communications, Culture*, Access to Knowledge & Information
	Individuals	Leaders*, Implementation Facilitators, Innovation Recipients, Capability*
3: Existing tools as a means to normalization and collaboration	Innovation	Relative Advantage, Adaptability, Trialability
	Outer Setting	Partnerships & Connections*
	Inner Setting	Structural Characteristics*, Communications*, Tension for Change, Compatibility
	Individuals	Leaders*, Implementation Facilitators, Innovation Deliverers, Opportunity
	Implementation Process	Teaming, Assessing Needs, Assessing Context, Planning, Engaging, Reflecting & Evaluating
4: Supervisors as central actors in a challenging task	Outer Setting	Critical Incidents
	Inner Setting	Structural Characteristics*
	Individuals	Leaders*, Implementation Facilitators, Innovation Deliverers, Capability*, Opportunity

CFIR: Consolidated Framework for Implementation Research^37^

* CFIR constructs with the most citations (*n* = 7–14).

### The acknowledgment and validation of employees’ emotions and experiences as a solution

The first theme illustrates the participants’ views that implementing the model might evoke negative and contradictory emotions in employees, i.e., the innovation recipients, due to the divide between objective assessments and subjective experiences. While participants recognized the legitimacy of employees’ symptoms and emotional reactions, they considered them disproportionate to the objective findings from building assessments. Acknowledging and validating employees’ experiences were seen as a potential solution. As we can see from Table 2, this theme was overarching in the Innovation domain, the Inner Setting domain, and the Outer Setting domain, covering several constructs.

The recognition and validation of employees’ emotions and experiences emerged as a key solution to bridging the gap between objective and subjective perspectives. Indoor environment issues were seen inherently as evoking emotional responses, such as fear and confusion, which add complexity to innovation efforts and influence the implementation of new models. Participants highlighted that acknowledging employees’ fears—often tied to concerns about illness—can prevent these fears from escalating rapidly at the mere mention of potential indoor environment issues. The following quote exemplifies how, by addressing “racing thoughts,” participants acknowledge that employee concerns can be amplified, and validating these emotions can prevent imagined scenarios from escalating beyond what is reasonable for the situation.

Often, it [a potential indoor environment problem] causes fear and raises questions such as ‘Is it possible for me to develop asthma in this situation?’ When you hear about some kind of indoor environment problem at the workplace, these thoughts can start racing. (Health and Safety Representative)

Workplace actors encounter significant challenges when addressing indoor environment issues, particularly in managing the strong emotional responses from employees, which necessitate careful attention and consideration to navigate effectively. The following quote illustrates how the work environment manager acknowledges the stress and complexity of these situations, using the expression “to put it nicely” to convey the gravity of the challenges faced. This acknowledgment is reinforced by framing it as shared knowledge with a researcher familiar with previous projects at the workplace, highlighting the importance of addressing the employees’ worries.

And, [researcher's name], you have attended our meetings in [city's name] and have likely witnessed our [employees’] responses, so you are aware of how critical and challenging the situation can be, to put it nicely. (Work Environment Manager)

Participants viewed that the gap between employees’ subjective experiences of symptoms linked to the building and the results of indoor environment quality assessments challenged the addressing of the employees’ emotions. This lack of causal evidence between symptoms and building-related factors was seen to leave employees feeling dismissed. Participants noted that contemporary researchers have shifted their understanding from solely building-related causes to a multifactorial perspective, meaning that the knowledge environment in the Outer Setting has changed, and must also change in the Inner Setting. In the following quote, the occupational safety manager acknowledges that the perceived etiology of symptoms might differ from the actual causes, while affirming the reality of the symptoms themselves. This approach highlights the importance of validating employees’ experiences and integrating multifaceted insights into addressing indoor environment issues.

Experts have suggested what they believe to be the etiology causing these [non-specific symptoms] in these situations, but the symptoms are nevertheless real. So, if I am sad, someone else can’t come and say that I’m not, or if I have respiratory symptoms, that is my experience of the matter, and nobody can deny it. And that is why starting from a point of accepting these [symptoms] as they are described will be the foundation for building trust as, at least in our organization, the mistrust has been tangible before. (Occupational Safety Manager)

### Reliable information and detailed facts as the key to managing work ability support

The second theme raises the topic of reliable information, by distinguishing between dividing “right” and “wrong” knowledge. Participants considered this a significant issue regarding health complaints associated with indoor environments. Reliable information, or “facts,” was viewed as a tool for managing these situations and fostering trustworthy communication. Scientific and objective knowledge was constructed as the “right” form of knowledge, providing reliable information and detailed facts to support work ability and the implementation of the model. Table 2 shows how this theme was overarching in the Innovation domain, the Inner Setting domain, the Outer Setting domain, and the Individuals domain, covering several constructs.

Participants considered that consistent and reliable information is crucial in the context of managing work ability support amid complex indoor environment-related health complaints. They noted that publicly available information (Outer Setting) and in-organization knowledge (Inner Setting) often lack consistency. Reliable, research-based information was highlighted as essential, as operating without scientifically validated data can lead to challenges. Participants suggested that reliance on non-research-based sources by employees or external experts might lead to conflicting views and impede effective decision-making, underscoring the need for accurate information to manage work ability support effectively.

So that we [implementation team] can direct them [supervisors and employees] to the right source because, with these indoor environment issues, there are a lot of things that arise from different sources, not necessarily based on research. And it is also difficult for us to rely on information that is not based on research, even though somebody else would rely on that, producing opposed arguments in the situation. (Work Environment Manager)

As demonstrated in the previous quote, guiding supervisors and employees to research-based sources to provide reliable facts was considered a crucial task for the implementation team (Individuals Domain). Researchers from the ongoing project and their organization were seen as sources of trustworthy information (Innovation Domain). Utilizing this “fact-based” information and the work ability support model, participants considered they could manage even the emotional aspects of these situations, emphasizing the importance of reliable data in effectively addressing work ability challenges.

And I hope that with the help of this material we asked for and got [from the researchers], we can create a good, fact-based model that dispels the suspicions between different actors. (Work Environment Chief)

And I see the importance of this communication that you mentioned, ensuring that the work community gets the exact message about the situation. It can easily cause fear and a certain kind of reaction if there's information about us having a problem related to our indoor environment. So, with a large amount of information or as exact an amount of information as possible, we can manage this too and support the work ability of the entire work community. (Work Ability Support Specialist)

Participants considered the accuracy of information as so important that they preferred systemic, structured discussions in workplace meetings over informal conversations. As the following quote illustrates, systematically providing employees with opportunities to discuss the situation in formal settings, such as staff meetings, was considered best practice (Inner Setting). Informal “coffee table” conversations were dismissed as “uninformed chatter,” suggesting they often contain incorrect information, which is seen as inferior to the accurate facts shared in employer-organized meetings.

So, this [systematic conversations in meetings] would probably relieve the situation, and the staff would be able to discuss it and talk through whether we’re doing well or poorly. If something comes up, it will be driven forward. And there won’t be uninformed chatter at coffee tables, to put it coarsely. (Work Environment Manager)

### Existing tools as a means to normalization and collaboration

The third theme highlights participants’ views on their organizations’ existing tools as beneficial for implementing the model. It also demonstrates how participants considered the existing tools and practices to have the potential to normalize indoor environment issues, thus enhancing stakeholder collaboration and reduce related tensions. Table 2 shows how this theme was overarching in all domains and covered multiple constructs across the CFIR.

However, these models and practices did not usually cover situations where employees reported health complaints attributed to indoor environments. Instead, these situations were commonly addressed with a building-oriented approach focused on continuous improvements of the building instead of early work ability support.

Leveraging existing tools was seen as a way to facilitate implementation, allowing the model to be integrated into established workplace practices (Innovation Domain, Inner Setting). The participants considered their own organizations having some form of work ability support models and practices in place. However, they viewed that these models typically did not address situations where employees reported health complaints related to indoor environments. Instead, such situations were often tackled with a building-oriented approach, emphasizing ongoing improvements to the building rather than early work ability support, underscoring the need for normalization and collaboration in addressing health complaints.

It seems to me that we have really good models here at the [Work Organization's name], but they mostly start from the point where there's already a defect in the building. And we've always waited until, whether they complain or not. (Occupational Safety Specialist)

Participants constructed normalizing health complaints related to indoor environments as essential and achieved by integrating the model into existing practices. Unlike other scenarios requiring work ability support, the complexity of indoor environment-related issues was emphasized (Innovation Domain). As the following quote illustrates, participants noted that these situations are often treated as anomalous or “made exceptional,” suggesting an element of exaggeration or dramatization, highlighting the need for normalization and collaborative efforts to address such challenges effectively.

It may be that this will become similar to any other issue so that these [situations] wouldn’t have to be made exceptional all the time. The same measures should be used, and we should be able to talk about this. (Occupational Safety Specialist)

In addition to utilizing familiar work ability support tools and practices, reframing indoor environment issues was constructed as a key strategy for achieving normalization and fostering collaboration. For instance, renaming indoor environment groups as work environment groups was mentioned as a way to integrate these issues into the broader context of workplace concerns, reducing their special status. This reframing aimed to make indoor environment issues “just one part” of the overall work environment framework, thereby diminishing their perceived exceptionalism and encouraging a more collaborative approach to addressing them.

Our teams managing indoor environment groups have developed into work environment groups, where they think about these issues more broadly. Indoor environment issues still form one of the main topics, of course, it doesn’t really vanish and probably never will. It's just one part of the overall work environment comfort and the work environment itself. (Work Environment Manager)

### Supervisors as central actors in a challenging task

The fourth theme shows how the participants considered supervisors as crucial in implementing the intervention, yet acknowledging the challenges due to potential controversies and required skills and resources. Supervisors were seen as central to work ability support processes, and the model was proposed as a tool to mitigate conflicts regarding work ability support in indoor environment situations. Table 2 illustrates how this theme was overarching in the Inner Setting domain, the Outer Setting domain, and the Individuals domain, covering several constructs (particularly in the Individuals domain).

Participants considered supervisor's role crucial in implementing and executing the intervention. They emphasized that supervisors already play a key role in work ability support, although their focus has traditionally been on issues unrelated to indoor environments. The model would necessitate that supervisors enhance their skills and knowledge to effectively support employees’ work ability in complex and potentially controversial situations involving indoor environment challenges, highlighting their pivotal role in navigating these demanding tasks.

I was also thinking about whether our supervisors, who are in key positions, will understand how and have the ability to operate according to our indoor air model and the work ability support model connected to it in the future. (Occupational Safety Manager)

I was thinking that it's important that the supervisor's area of responsibility and number of employees allow for realistically observing changes in work ability. For example, if [a supervisor] has seventy employees, that's a lot of work to keep up with potential changes in work ability [the work ability of employees]. So, there's quite a big responsibility on supervisors in this model. (Health and Safety Representative)

Given the prevailing building-oriented approaches and the special status of indoor environment issues, participants viewed that supervisors might not be well-versed in work ability support for these situations. Traditionally, the responsibility for finding solutions has fallen predominantly to technical and construction experts, as well as higher-level leaders involved in indoor air working groups (Inner Setting, Outer Setting, Individuals Domain). This reflects a significant shift in organizational culture, underscoring the need for supervisors to develop competence and understanding of the complex, multifactorial nature of indoor environment challenges, positioning them as central actors in this demanding task.

This is quite a big change. You see, very few of our supervisors have actually been involved in solving this indoor air problem. For example, our indoor air working groups have mostly included higher-level unit managers. So, most of the supervisors, like team leaders, haven’t been involved in this much at all up until now. And now the plan is to get this entire group of supervisors involved. (Upper-level manager)

Participants viewed that the coherence and consistency provided by the model would empower supervisors to effectively handle situations concerning indoor environment health complaints. As the following quote illustrates, it was suggested that the model was undeniably the correct approach for addressing all work environment issues, reflecting confidence in its comprehensive ability to resolve challenges. This implies that alternative approaches would be considered less favorable, and in instances of differing opinions on necessary actions, the model would serve as a guiding framework for decision-making, emphasizing the supervisor's central role in navigating complex tasks.

All questions related to the work environment should be addressed using this model, and there is an opportunity to tackle more challenging work environment issues with it. For example, if the team wants a certain kind of measure or has a specific problem, [the supervisors] could use this model and would know how it progresses. It would be a controlled, managed process that leads to the goal. And all parties would know that this is the way to advance and handle this issue, making it clear for the entire administration and all participants. (Work Environment Manager)

## Discussion

This study explored the workplace actors’ views of implementation regarding a model for supporting work ability related to health complaints from indoor environments. Participants emphasized the acknowledgment and validation of employees’ emotions and experiences, the significance of reliable information and detailed facts, normalization and collaboration using existing tools and supervisors as central actors in the challenging task of both implementation and the work ability support. The findings emphasize the necessity to address underlying controversies and tensions and to integrate diverse perspectives, experiences, and opinions for the implementation to succeed.

Our four developed overarching themes covered several domains and constructs within the CFIR framework, crystallizing the major contextual entities that must be widely considered when implementing the model. The themes share common elements of potential controversy and significant emotional burden related to health complaints associated with indoor environments. These arise from the complex, subjective and multifactorial nature of the health complaints, which the model focuses on, along with the necessity of adopting a multi-perspective approach in its implementation to effectively address associated work ability challenges. Previous research from healthcare contexts shows that a multi-perspective approach for interventions addressing subjective health complaints is recommended.^23,25,27,28^ At the workplace level, previous research regarding subjective health complaints and work ability support does not exist to our knowledge. However, combining multiple perspectives has proven effective in implementing programs such as mental health initiatives in the workplace.^44,45^ Our findings extend previous research by emphasizing that, alongside multi-perspective approaches, addressing potential controversies and emotional factors is crucial in implementing work ability support models for complex health issues.

Our findings suggest that a primary source of potential controversy lies in the necessity to integrate subjective and objective knowledge when implementing interventions for multifactorial health complaints and related work ability. This stems from the necessity of validating employees’ subjective experiences while operating according to objective information deriving from scientific research and investigations of the building. However, connecting objective and subjective knowledge to create practices for individuals with subjective health complaints is not always easy and may cause friction.^46^ Regarding subjective experiences, previous research on indoor environment settings indicates that ignoring employees’ emotional reactions can lead to workplace conflict and mistrust.^47,48^ Furthermore, in healthcare contexts, professionals’ positive attitudes and empathy have been shown to facilitate the implementation of interventions for patients with subjective health complaints.^49^

On the other hand, the need for reliable and objective information is understandable and supported by previous research. Firstly, the reliability of the innovation source and supporting scientific knowledge are crucial for successful implementation.^37^ Secondly, knowledge and beliefs about innovation significantly impact implementation and relate to numerous constructs within the CFIR.^37^ For example, the knowledge and beliefs of management, innovation deliverers (e.g., healthcare professionals or workplace supervisors), and innovation recipients (e.g., patients or employees) are vital for successful implementation.^49,50,51^ Lastly, the inherent uncertainty and complexity of subjective and multifactorial health complaints, which pose challenges even in healthcare settings,^52^ underscore the need for precise information.

One problem in integrating subjective and objective knowledge is which knowledge to prioritize. The implementation teams in our study tended to stress the importance of science-based knowledge and its sharing from top to down, that is, passing from implementation facilitators and supervisors to employees. While reliable, science-based information is essential for implementation, portraying this information as “right” compared to other types of information may impede the implementation process. In this way, science-based knowledge becomes framed as an epistemic authority,^53^ despite its inherent complexity and frequent scientific disagreements, which can confuse the public.^54^ Framing science-based information as “right” also implies the existence of “wrong” information, potentially conflicting with the need to acknowledge and validate employee concerns. Integrating scientific knowledge with subjective experiences is challenging and may require accommodating different opinions and finding transparent approaches to resolve conflicts.^46^

Many workplaces already have established practices for supporting work ability, as did the participating organizations in this study. Previous implementation research shows that compatibility with workflows and existing practices, along with the adaptability of innovations, is essential.^37,49^ Thus, integrating a work ability support model addressing subjective and multifactorial health complaints into the existing practices enhances the implementation. Our findings contribute to this body of knowledge by suggesting that leveraging existing tools and practices to address complex, environment-related health issues in the workplace can facilitate the normalization of indoor environment situations, thereby transforming extraordinary circumstances into ordinary ones. Normalization can be understood as an institutionalized process that makes extraordinary situations ordinary through, for example, reframing or adaptation.^55^ In the indoor environment context, this would involve reframing psychological aspects, such as employees’ emotions and experiences, as acceptable. This aligns with previous research noting that the needs of innovation recipients are crucial for implementation outcomes.^56,57^ However, transitioning from the traditional building-oriented perspective requires time, resources, and a shared understanding within the organization.

Supervisors are pivotal in implementing work ability support measures, linking employees with employer representatives and healthcare providers.^9,58^ Regarding addressing subjective and multifactorial health complaints and related work ability, supervisors’ already demanding roles intensify. Our findings suggest that they would be required to validate employees’ emotions, integrate subjective and objective knowledge, and reframe indoor environment situations as routine work ability support cases. Previous research underscores that successful implementation of interventions for sensitive issues, such as mental health or multifactorial health complaints, requires innovation deliverers to develop soft skills and foster trustful relationships to effectively manage recipients’ emotions.^49,59,60^ Our findings align with this, emphasizing the necessity for supervisors to gain understanding of indoor environment-related health complaints and skills for communication.

Our findings underscore the critical role of communication in implementation, which is not novel. However, we extend this importance beyond mere information exchange and best practices within organizations. Effective communication is crucial for organizational sensemaking and learning during innovation adoption, yet remains challenging.^61^ The complexity of subjective and multifactorial health complaints further complicates communication. Implementing a multi-perspective, potentially controversy-inducing work ability support model requires integrating diverse perspectives, experiences, and opinions. Consistent with Manojlovich et al.,^62^ we propose that adopting a constructionist perspective on communication can enhance this process. This approach views communication as a means of developing shared understanding and co-constructing knowledge. Our study's practical implications, summarized in Table 3, center on communication as a means to achieve shared understanding

**Table 3.** Practical implications for implementing a work ability support model related to health complaints attributed to indoor environments.

Implementing an intervention addressing an intricate health phenomenon requires a multi-perspective approach.
A shared understanding of these aspects is crucial among all stakeholders.
A narrower, building-oriented focus may still prevail and can hinder the implementation of work ability support.
Communication should be viewed as a process of developing shared understanding and co-constructing knowledge throughout the implementation.
Situations may raise concerns and create both a lack of trust and a risk of social stigmatization.
Allowing for different opinions and integrating subjective and objective knowledge is important.
Normalizing health complaints related to indoor environments as one instance among others of needing work ability support.
Organizations have already tools for work ability support that can also be used for indoor environment issues.
Normalization may require time, dialogic communication, and resources.
Supervisors, as key actors, face a challenging task of integrating subjective and objective knowledge and acknowledging the employees’ concerns during the implementation.
The competencies and skills of supervisors vary regarding indoor environment issues and work ability.
Supervisors need training, skills, and support when implementing and acting in line with the new model.

### Strengths and limitations

Our study has both strengths and limitations. The follow-up period was limited for the implementation process, as indicated by the few excerpts falling into the Implementation Process domain (see Table 2). Further research is needed to explore the complete implementation process. Despite the small size of our data, the recordings were very rich and focused, with minimal discussion of issues irrelevant to implementation. Although the workshops were facilitated and included presentations from the facilitators (see Table 1), we exclusively recorded the discussion sections of workshops, which were loosely facilitated, allowing for a wide-ranging discussion to arise from the participants. Using RTA with a constructionist approach allowed for an in-depth analysis of the existing data, concentrating on what the participants constructed as meaningful rather than imposing the researchers’ perspectives.^42^ A strength of the study was that participants were experienced and motivated in developing work ability support and resolution processes for indoor environment issues, ensuring engaged involvement. Prior participation in the model development by two organizations further enriched the data's depth and quality. Additionally, the organizations represented three very different public industries.

## Conclusions

This study contributes to the implementation research of complex interventions, especially those focused on subjective health complaints, within the context of a workplace and characterized by public debate and diverse perceptions. As the overarching themes and their encompassment of CFIR show, tensions arising from the complexity and controversiality of the phenomenon may affect widely different aspects of implementation. Based on our findings, we suggest that implementing a complex intervention addressing an intricate health phenomenon requires viewing communication as a process of developing shared understanding and co-constructing knowledge throughout the implementation process and all its dimensions, instead of a merely transactional process.
